# Extensive Variation in the Density and Distribution of DNA Polymorphism in Sorghum Genomes

**DOI:** 10.1371/journal.pone.0079192

**Published:** 2013-11-12

**Authors:** Joseph Evans, Ryan F. McCormick, Daryl Morishige, Sara N. Olson, Brock Weers, Josie Hilley, Patricia Klein, William Rooney, John Mullet

**Affiliations:** 1 Department of Biochemistry and Biophysics, Texas A&M University, College Station, Texas, United States of America; 2 Department of Horticulture, Texas A&M University, College Station, Texas, United States of America; 3 Department of Soil and Crop Sciences, Texas A&M University, College Station, Texas, United States of America; CNR, Italy

## Abstract

Sorghum genotypes currently used for grain production in the United States were developed from African landraces that were imported starting in the mid-to-late 19^th^ century. Farmers and plant breeders selected genotypes for grain production with reduced plant height, early flowering, increased grain yield, adaptation to drought, and improved resistance to lodging, diseases and pests. DNA polymorphisms that distinguish three historically important grain sorghum genotypes, BTx623, BTx642 and Tx7000, were characterized by genome sequencing, genotyping by sequencing, genetic mapping, and pedigree-based haplotype analysis. The distribution and density of DNA polymorphisms in the sequenced genomes varied widely, in part because the lines were derived through breeding and selection from diverse Kafir, Durra, and Caudatum race accessions. Genomic DNA spanning *dw1* (SBI-09) and *dw3* (SBI-07) had identical haplotypes due to selection for reduced height. Lower SNP density in genes located in pericentromeric regions compared with genes located in euchromatic regions is consistent with background selection in these regions of low recombination. SNP density was higher in euchromatic DNA and varied >100-fold in contiguous intervals that spanned up to 300 Kbp. The localized variation in DNA polymorphism density occurred throughout euchromatic regions where recombination is elevated, however, polymorphism density was not correlated with gene density or DNA methylation. Overall, sorghum chromosomes contain distal euchromatic regions characterized by extensive, localized variation in DNA polymorphism density, and large pericentromeric regions of low gene density, diversity, and recombination.

## Introduction

Genome sequencing is being used to characterize genome-wide patterns of genetic variation and inheritance, to help identify regions of genomes under selection, and to aid in the discovery of SNPs and alleles associated with quantitative trait loci (QTL) [1]–[5]. Sequences from target genomes can be readily aligned to reference genome sequences, enabling the identification of sequence variants [6]. This re-sequencing approach identified over a million sequence variants between closely related rice cultivars and between sorghum genotypes, including gene-coding variants that could affect function [5], [7]. While this technique may not currently be cost-effective for plants with very large genomes that lack reference sequences, it has great potential for sorghum (∼818 Mb), a genetic model for C4 grass species, and an important drought tolerant grain, forage, and energy crop [8], [9], [10].

Whole genome re-sequencing of key breeding lines can provide valuable information regarding the lineage of a cultivar and the origins of different portions of the cultivar’s genome [3], [11]. Moreover, the genome sequences can be compared to help identify regions that have undergone selection [10], [12], [13]. This is especially relevant for cultivated sorghums in the United States because the elite sorghums used in hybrid grain production are products of domestication in Africa ∼4,000 to 6,000 years ago, followed by many subsequent generations of breeding and selection in different locations [14]. Systematic grain sorghum development in the United States began with the introduction of sorghum landraces and exotic accessions from Africa beginning in the mid-to-late 19^th^ century, followed by selection of genotypes exhibiting high grain production with reduced height, early maturity, increased grain quality, adaptation to local environments, and resistance to lodging, diseases, and pests [9], [15], [16]. Sorghum accessions used in grain production are very diverse and were derived from the five sorghum races that arose following divergence of sorghum from rice ∼50 MYA [17]. Sorghum races are prevalent in different regions of Africa, reflecting the diverse agro-eco-environments to which they are adapted: Kafir in southern Africa, Guinea in west Africa, Caudatum in north-central Africa, Durra in Sudan/Ethiopia/Egypt and Bicolor, the most ancient race, in the northeast quadrant of Africa, the center of origin for sorghum [14].

The *Sorghum bicolor* genome is comprised of ten chromosomes spanning ∼818 Mbp of DNA [18]. Sorghum chromosomes contain large pericentromeric regions covering ∼50% of the genome that are characterized by low gene density and very low rates of recombination [19]. Euchromatic DNA that spans the outer portion of each chromosome arm has higher gene density (∼1 gene/12 Kbp) [19]. The current ‘reference’ BTx623 genome sequence assembly (Sb1) spans 720 Mbp of DNA forming 10 pseudomolecules, corresponding to the ten sorghum chromosomes, plus numerous small “super-contigs” that were not integrated into the reference genome sequence [20].

In this study, sequences from the genomes of Tx7000 and BTx642 were obtained and aligned to the BTx623 reference sequence to aid in the identification of DNA polymorphisms. Digital Genotyping (DG) [21] and haplotype tracing through pedigrees were used to identify the origins of DNA in each genome and portions of the three sequenced genomes that are identical, or nearly identical, by descent. The overall analysis revealed extensive variation in DNA polymorphism density distribution across the sorghum chromosomes.

## Results

### Genotype Selection and Analysis of Genetic Relationships

Tx7000 and BTx642 were selected for genome re-sequencing and comparison to BTx623 for several reasons. Tx7000 (Caprock), released in 1941, was an elite variety used for grain production in the United States in the 1940–50s prior to the development of hybrids [16]. Tx7000 is a source of pre-anthesis drought tolerance and was used as an elite R-line for production of early grain sorghum hybrids (e.g. Tx660) in the 1950s [22]. BTx642 was selected for sequencing in part because this genotype is the most important source of post-anthesis stay-green drought tolerance in sorghum [22]–[24]. BTx642 has been used in breeding programs in the United States, Australia, India and other parts of the world to improve the drought tolerance of grain sorghum [24]. In addition, a recombinant inbred line (RIL) population derived from BTx642 and Tx7000 is available and has been used to map QTL for the post-flowering drought tolerance stay-green trait and other traits [23], [25]. Therefore, genome sequences of BTx642 and Tx7000 will aid the identification of genes/alleles that contribute to numerous important plant traits. Moreover, BTx623, Tx7000, and BTx642 are derived from accessions classified as Kafir, Durra, and Caudatum, providing an opportunity to identify and compare DNA polymorphisms that distinguish these sorghum races.

**Figure 1:**
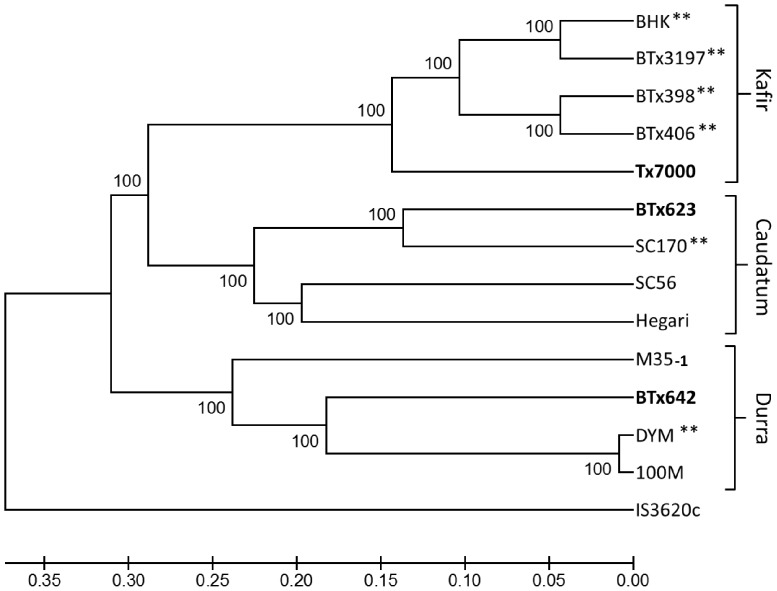
Genetic relationships among sorghum genotypes. Sorghum lines analyzed include the sequenced genotypes BTx623, Tx7000, BTx642 (bold), progenitors (**) of the sequenced genotypes Blackhull Kafir (BHK), Dwarf Yellow Milo (DYM), SC170, BTx398, BTx3197, BTx406, and lines representing sorghum races (Guinea: IS3620C; Caudatum: SC170, SC56, and Hegari; Kafir: BHK; Durra: M35-1, DYM, and 100M). This dendrogram was constructed using UPGMA.

The genetic relationships between BTx623, BTx642, Tx7000, and their progenitors in the U.S. grain sorghum breeding program, and other representative genotypes were analyzed using sequence information derived by Digital Genotyping, a genotyping by sequencing method developed for sorghum [21]. DG-sequences 37 bp or 72 bp in length, which flank *Ngo*MIV restriction sites in the sorghum genome, were collected from many of the genotypes in the pedigrees of BTx623, Tx7000 and BTx642 [28]. Approximately 14,000 unique DG-sequences derived from different locations in the sorghum genome, spanned polymorphisms that distinguish the genotypes analyzed. These polymorphic DG-sequences (DG-markers) were used to characterize the overall genetic relationships among the genotypes [26]. Unweighted pair group method with arithmetic mean (UPGMA) hierarchical clustering [27] was used to generate a dendrogram representing the genetic relationships among the genotypes analyzed (Fig. 1). This analysis showed that BTx642 clusters with other Durra genotypes (e.g., M35-1, Dwarf Yellow Milo, and 100M), and Tx7000 clusters with Kafir genotypes such as Blackhull Kafir, one of the parents used to construct Tx7000 (Fig. 1). BTx623 clusters with SC170, one of the parents used to develop BTx623 and SC170 clusters with Caudatum genotypes (e.g. Hegari and SC56). The genetic relationships determined using DG-marker data were consistent with prior analysis of a subset of these genotypes with SSR and AFLP markers [28], [29].

### Genome Sequencing and DNA Polymorphism Discovery

**Figure 2:**
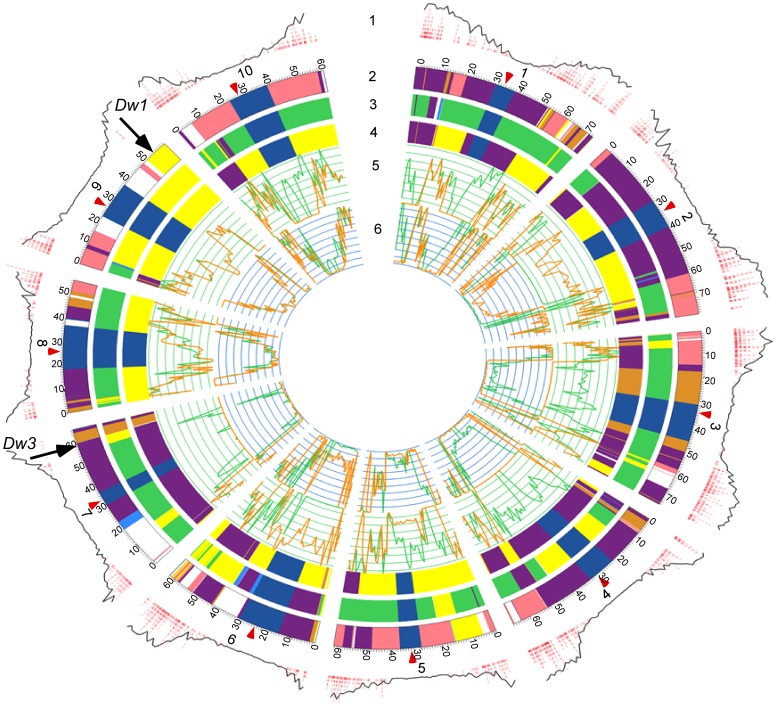
Distribution of DNA polymorphisms found in comparisons of BTx642/BTx623 and Tx7000/BTx623 in the ten sorghum chromosomes. The outside track is composed of two parts: gene density (outer black line) and locations of unique non-methylated DG-sequences (red bars, outer track). Next, tracks 2, 3, and 4 representing BTx623, BTx642 and Tx7000 chromosomes #1–10, colored to indicate DNA origin, are shown; purple represents Blackhull Kafir, red represents IS12261 (Caudatum), yellow represents Durra (Milo), green represents IS12555 (Durra), light brown represents BTx3197, and dark blue signifies pericentromeric regions, while white represents DNA of unknown origin. The fifth track represents SNP density (SNP/Mbp) and the sixth track, INDEL density (INDELs/Mbp). For both of the inside tracks, comparison between BTx642 and BTx623 is shown in green while comparison between Tx7000 and BTx623 is shown in orange. Locations of *Dw1* (SBI-09) and *Dw3* (SBI-07, 58.6 Mbp) are indicated, and centromeres are denoted with red arrowheads.

Genome sequences from BTx642 and Tx7000 were acquired using Illumina TruSeq reagents and a HiSeq2000 sequencing system, aligned to the BTx623 reference sequence, and SNPs and INDELs that distinguish the genotypes were identified (Fig. 2, Tracks 5, 6). Paired-end sequencing generated 137 million reads, with an average read length of 94 bases for a total of 12.9 Gbp that passed quality control. To obtain deeper coverage, additional 100-base single-end reads were obtained from the same DNA libraries. Single-end sequencing generated 267 million sequences (98-base reads) and 26.3 Gbp of sequence information for a combined total of 39.2 Gbp from the BTx642 and Tx7000 genomes. Sequences from each genotype were aligned to the BTx623 genome sequence using the *Map Reads to Reference* function of the CLC Genomics Workbench. Approximately 190 million reads (120 million single-end reads, 70 million paired-end reads) from each genotype aligned to the BTx623 reference genome sequence, while approximately 33 million reads from each genotype did not align to the reference sequence, even when the stringency of mapping criteria was reduced. This indicates that the non-aligned reads likely originate from DNA that is not included in the reference genome sequence such as centromeric regions of the genome where the assembled sequence data in the reference genome may have limited coverage, genetic material that was not anchored to the reference genome, the mitochondrial genome, the chloroplast genome, or another source of DNA. Overall, the sequences obtained from Tx7000 and BTx642 that were aligned to the reference sequence provided a 25-fold average depth of sequence coverage across the genomes.

### Sequence Quality Assessment

**Figure 3:**
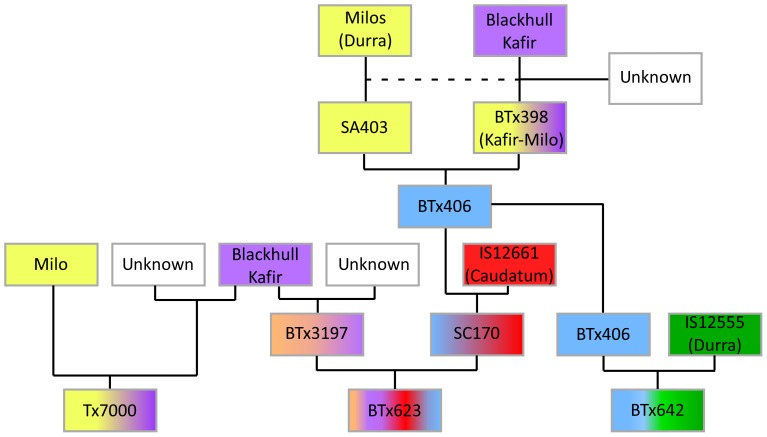
Pedigrees of BTx623, Tx7000 and BTx642. Genotypes based on parentage are color coded as follows: Blackhull Kafir (purple), Milo-Durra genotypes (yellow), IS12661 (Caudatum) (red), IS12555 (Durra) (green), BTx3197 (orange-brown) and BTx406 (blue). Digital Genotypes were obtained from BTx623, Tx7000, BTx642, BTx406, SC170, BTx3197, Blackhull Kafir, BTx398 and Milos (Dwarf Yellow Milo) and used to trace haplotypes through the pedigrees.

The quality of DNA variants identified by re-sequencing was analyzed by comparison to DG-marker sequences validated through deep sequencing and genetic mapping [30]. BTx642, Tx7000 and 90 RILs (F_12_) derived from these parental lines were subjected to Digital Genotyping [21]. For each sample, 72 bp sequences adjacent to *Ngo*MIV sites of digestion were obtained at a minimum of 5-fold depth (average depth per DG-sequence per line of ∼15X). DG-sequences obtained from BTx642 and Tx7000 that mapped uniquely to one location in the sorghum genome were compared to identify DG-markers. The sequences of 1462 DG-markers were obtained from the 90 RILs and the DG-marker information was used to construct a genetic map that spanned 1130 cM across the ten sorghum chromosomes (Fig. S1). DG-marker order in the genetic map was consistent with the physical order of DG-sequences in the reference genome sequence. The 1462 DG-validated, 72 bp sequences from each genotype were compared with the corresponding sequences obtained from the alignment of short read sequences from Tx7000 and BTx642 to the BTx623 reference sequence. The analysis showed that all of the DG-sequences spanning SNPs that differentiate Tx7000 or BTx642 from BTx623 were identified in the re-sequenced genome assemblies. This indicates that the genome sequences obtained by re-sequencing provide good coverage of the unique sequence portion of the sorghum genome accessed by DG-analysis. Of the 1462 DG-sequences analyzed (102,000 bp), 1356 Tx7000 sequences and 1293 BTx642 sequences aligned perfectly with the corresponding sequences obtained by whole genome re-sequencing and alignment (∼90.6%). Examination of the remaining 9.4% of the DG-sequences obtained from genome re-sequencing revealed that nearly all contained INDELS that did not align perfectly with, or were not present in, the validated DG-sequences that spanned INDELs. The most likely explanation for the INDEL errors is lack of fidelity in alignment of short reads from the whole genome to regions containing sequence repeats. Fidelity of re-sequencing and assembly was also assessed using larger scale comparisons. For example, DG-marker analysis identified several regions in the three genotypes that were identical or nearly identical by descent (IBD) (see below). Comparison of these IBD regions after re-sequencing and assembly showed minimal DNA polymorphism across these haplotypes (Fig. 3, described below). It was also possible to determine the reproducibility of independent re-sequencing and assembly by comparing results from the same haplotypes present in two genotypes. For example, the region of SBI-02 from 75–76 Mbp in Tx7000 and BTx642 was derived from BTx3197 and differed from BTx623 (see below). The SNP profiles that distinguish each of the re-assembled sequences from BTx623 were very similar (Fig. S2).

### DNA Polymorphism Density Distribution in Chromosomes

Comparison of the sequences of Tx7000 and BTx642 to BTx623 identified more than 2.8 million DNA single nucleotide polymorphisms (SNPs) and small insertion-deletion polymorphisms (INDELs). The analysis identified 1.2 M SNPs and 120,969 INDELs that distinguish the Tx7000 sequence from BTx623, and 1.6 M SNPs and 152,836 INDELs that distinguish the BTx642 sequence from BTx623. Approximately 86% of the SNPs were located in intergenic DNA, with the remaining 14% in annotated genes including 7% in introns, 6% in coding regions, and 0.9% within UTRs (Table 1). In coding regions, 66% of the SNPs did not change amino acid sequences, 33% caused an amino acid change, and 1% resulted in a premature stop codon or stop-lost condition. While the proportion of premature stop and stop-lost variants was small when compared with all of the SNPs identified, 296 were identified that distinguish Tx7000 from BTx623, and 360 were identified that distinguish BTx642 from BTx623. Overall, nearly 1% of annotated genes contain variants that could affect gene function. Approximately 83% of the INDELs were located outside of annotated genes. Of the INDELs located within genic regions, ∼12% were in introns, ∼2% in coding regions, and ∼3% were located in 5′ UTR sequences. INDELs present in coding regions caused frameshift coding variants, premature stop codons, stop-lost variants, and amino acid substitutions (Table 1).

**Table 1:** DNA polymorphisms identified in comparisons of BTx642/BTx623 and Tx7000/BTx623 genome sequences.

SNPs	Intragenic	Intron	UTR	Coding	AA Change	Premature Stop	Stop Loss	Frame Shift
BTx642	1441069	111106	14764	62031	30342	296	66	N/A
Tx7000	1045805	89004	11733	50779	24908	226	70	N/A
INDELs								
BTx642	129334	18065	3281	2167	270	6	1	1814
Tx7000	101502	14605	2691	1901	346	2	2	1627

The distribution of SNPs and INDELs that distinguish BTx642 and Tx7000 from BTx623 was determined for each of the ten sorghum chromosomes (Fig. 2, Track 5 (SNPs), Track 6 (INDELs)). Extensive variation in the distribution of SNP and INDEL density was observed across the genome and within individual chromosomes in each comparison. Every chromosome had an extended region of relatively low SNP and INDEL density spanning the pericentromeric region consistent with results from genotyping analysis [17], [36]. The distribution of DNA polymorphism density across the gene rich euchromatic arms of chromosomes was highly variable, interrupted in a few places by extended regions of very low DNA polymorphism (Fig. 2). Some of this variation could be due to comparison of regions that are derived from different sorghum races, identical by descent, under selection, and/or modified by other factors that are examined in greater detail below.

### Pedigree and Haplotype Analysis

To better understand the DNA polymorphism density distributions shown in Figure 2, the pedigrees of BTx623, Tx7000, and BTx642 were constructed to help determine the origin of DNA in the sequenced genomes. BTx623 is an elite female line that has been utilized for hybrid grain production since the 1960s [31]. BTx623 was derived from a cross of BTx3197 and SC170 [28]. BTx3197 is a Kafir sorghum derived from Blackhull Kafir and an unknown double dwarf waxy Kafir genotype [16]. SC170 was derived from a cross of IS12661, a Caudatum accession, to BTx406, a short, early-flowering Kafir-Milo genotype, followed by a backcross to IS12661 with selection for short height and early flowering. This was done to convert IS12661 from a late-flowering, tall genotype to SC170, a short, early-flowering variety useful for grain production. The pedigree of BTx406 involves crosses among the Milo (Durra) genotypes Dwarf Yellow Milo, Double Dwarf Yellow Milo, Early White Milo, and Double Dwarf White Sooner Milo, creating the short, early flowering Milo genotype SA403 [32]. SA403 was then crossed to BTx398 (Martin), a Kafir-Milo genotype developed during the same period as BTx3197, to generate BTx406 [32]. The pedigree of Tx7000 (Caprock), a Kafir-Milo line, is not fully known [16]. This genotype was most likely derived from Blackhull Kafir (1890), the Durra sorghum Dwarf Yellow Milo (1906), and other, unspecified Kafir and Milo genotypes that were crossed and selected between 1920 and 1940 [16]. BTx642 is a BC_1_ derived line from a cross of IS12555 (Durra) to BTx406 followed by one backcross to IS12555 with subsequent selection for short height and early flowering (William Rooney, pers. communication) [28].

Polymorphism data from 8900 DG-markers was used to identify the origin of DNA in each of the sequenced genomes by tracing haplotypes through pedigrees to each of the sequenced genomes (Fig. 3) [28]. Haplotypes based on this analysis are shown in a circular diagram of the sorghum chromosomes that also displays gene density and location of DG-marker sequences used in the analysis (Fig. 2, Track 1: gene density, black line; DG-markers, red stacks). Haplotypes comprising the BTx623, BTx642, and Tx7000 genomes are shown in Tracks 2, 3 and 4, respectively, along with chromosome numbers, sizes (Mbp) and the approximate locations of centromeres (red arrowheads) (Fig. 2). The haplotypes of the genomes were color-coded based on the sorghum accession or genotype from which the haplotype originated in the pedigree (Fig. 2: Blackhull Kafir, purple; SC170 (Caudatum), red; Milo (Durra), yellow; IS12555 (Durra), green; BTx3197, light brown; BTx406, light blue; Unknown, white). Few DG-marker sequences were derived from portions of the pericentromeric DNA, therefore haplotypes in these regions were not assigned (Fig. 2: No data, dark blue). Where possible, DG-marker alleles from both parents were used to identify the origin of DNA in progeny derived from each cross in the pedigrees. For example, most of the DNA present in BTx623 could be traced back to BTx3197 or SC170. However, approximately 10% of the DG-markers in BTx623 could not be traced to either of these two genotypes (Fig. 2, Track 2, white blocks). One possible explanation for this observation is that the SC170 and BTx3197 lines genotyped in this study are more inbred than the original seed sources used to construct BTx623, resulting in the presence of short haplotypes in BTx623 not found in current versions of these genotypes. Alternatively, several closely related versions of SC170 and BTx3197 may have been generated, and genotyping was done on a variant of the line used to construct BTx623. In other cases, the genotype of only one of the parents of a line was available (e.g. BTx406 but not IS12555 for BTx642). In these cases, DNA from the genotyped parent was identified (e.g. BTx406) and the remaining portion of the genome with DG-alleles not derived from that parent was tentatively assigned to the other parent (e.g. IS12555). Most haplotypes spanned >50 consecutive markers, and many spanned hundreds of DG-markers, indicating that DNA was derived from one of the two parents involved in specific crosses in the pedigree. Some haplotypes spanned only a few markers, especially at the ends of chromosomes where recombination rates are high, and in pericentromeric regions where DG-marker density is low. These regions, and the junctions between haplotypes corresponding to breakpoints, were supported by fewer DG-markers, and therefore greater uncertainty is associated with their haplotype assignments. Overall, haplotypes assigned based on DG-marker data were consistent with the distribution of DNA polymorphisms identified by re-sequencing analysis. In some cases haplotypes of regions with limited DG-marker coverage could be determined by analysis of whole genome sequences and comparison of all SNPs/INDELs that distinguish BTx623, BTx642, and Tx7000.

Haplotype tracing through pedigrees revealed that DNA comprising the three sequenced sorghum genomes was derived during breeding primarily from six diverse sorghum accessions. All three genomes contained regions with haplotypes identical to Blackhull Kafir and Dwarf Yellow Milo (DYM) because these accessions are in the pedigrees of all three sequenced genotypes (Fig. 2, Tracks 2–4, purple and yellow blocks). There were a few haplotypes in common among BTx623, BTx642 and Tx7000: a DYM haplotype on SBI-09 (50.2–59 Mbp) spanning dwarfing locus 1 (*dw1, Sb-HT9.1*) [33], [34], a haplotype on SBI-07 (58.5–62.8 Mbp) spanning dwarfing locus 3 (*dw3*) [35], and smaller regions of SBI-01 (8–10 Mbp), SBI-02 (9–12 Mbp), SBI-04 (4–5.5 Mbp), and SBI-05 (13–15.2 Mbp). Haplotype tracing through pedigrees also explained the origin of several additional regions of low DNA polymorphism where haplotypes of the same origin (IBD) were being compared. For example, a central region of SBI-02 from ∼12.5–60 Mbp largely lacked DNA polymorphisms that distinguish BTx642 and BTx623 because DNA spanning that region in both genotypes originates from Blackhull Kafir (Fig. 2, Tracks 2 and 3). In contrast, extensive SNP and INDEL density variation between BTx623 (Blackhull Kafir haplotype) and Tx7000 (Milo-Durra haplotype) was observed across this same region of SBI-02 (Fig. 2, Track 2 vs. 4). SBI-03 from ∼15–59 Mbp shows a high density of DNA polymorphism between BTx642 and BTx623, and a very low level between Tx7000 and BTx623, because these latter genotypes share the BTx3197 haplotype across this region (Fig. 2). Overall, pedigree-based haplotype analysis of the three sequenced genomes allowed pairwise investigation of DNA polymorphism across large genomic regions that differed in haplotypes derived from the six diverse sorghum accessions involved in construction of BTx623, BTx642 and Tx7000. The analysis also enabled the identification of regions of the three genomes that are identical, or nearly identical by descent, explaining why these regions show minimal DNA polymorphism.

### Analysis of DNA Polymorphism Density Distribution in Sorghum Chromosome 2

**Figure 4:**
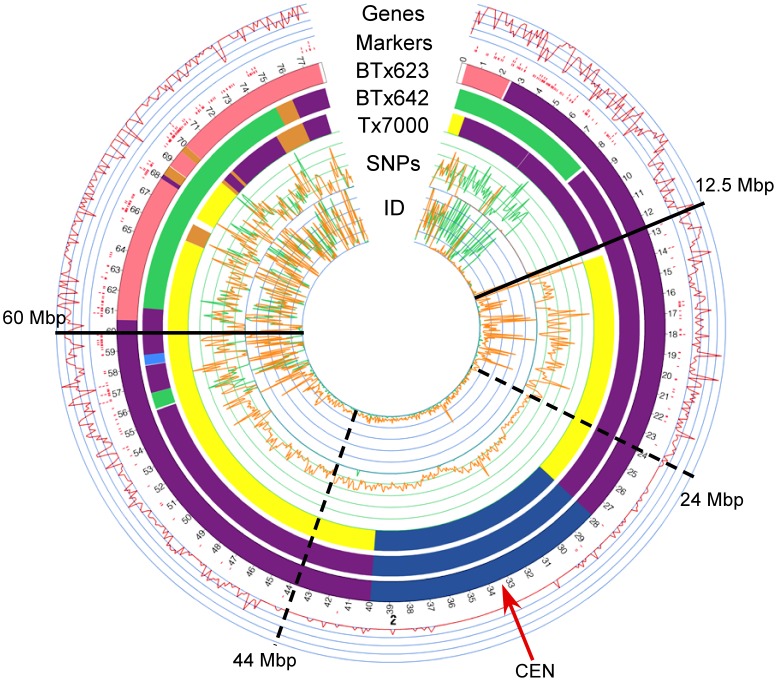
Distribution of genes and DNA polymorphism in sorghum chromosome 2. Beginning at the outside of the diagram, the tracks shown represent the following: gene density, DG-marker density, DNA haplotypes of BTx623, DNA haplotypes of BTx642, DNA haplotypes of Tx7000, SNP density, and INDEL (ID) density. For both SNP and INDEL density tracks, comparison between BTx642 and BTx623 is shown in green, while comparison between Tx7000 and BTx623 is shown in orange. Solid lines flank the region from 12.5–60.2 Mbp where the haplotypes of Tx7000 (Milo-Durra) and BTx623 (Kafir) differ. The region from 24–44 Mbp, demarcated with dashed lines, corresponds to the pericentromeric region of low gene density and recombination of SBI-02.

The genome-wide variation in SNP density distribution was investigated in greater detail on SBI-02 (Fig. 4). SBI-02 was selected for in depth analysis because this chromosome had a DNA polymorphism density distribution similar to other chromosomes and it lacked known agronomic QTL such as *Dw1, Dw3* and *Ma1* that are under strong selection in grain sorghum breeding programs [15], [36]. Euchromatic DNA spans the first ∼20–24 Mbp on the short arm of SBI-02 and the terminal ∼30 Mbp of the long arm of SBI-02 (Fig. 4, regions marked by dashed lines) [19]. The proximal ∼2.5 Mbp of the short arm of SBI-02 in BTx623, BTx642, and Tx7000 was derived from different sorghum races and shows a high level of DNA polymorphism. In contrast, DNA from 8.5–12.5 Mbp in all three genotypes was derived from Blackhull Kafir and showed minimal DNA polymorphism. The haplotypes of Tx7000 (Milo-Durra) and BTx623 (Blackhull Kafir) differ across the region of SBI-02 from ∼12.5 Mbp to ∼60.2 Mbp. Comparison of Tx7000 and BTx623 sequences across this region showed high levels of variation in SNP and INDEL densities from ∼12.5–24 Mbp, and lower levels and less variation in DNA polymorphism density in the heterochromatic pericentromeric region from ∼24–44 Mbp (Fig. 4). The level and frequency of DNA polymorphism increased again in the euchromatic portion of the long arm of SBI-02. Lower relative SNP and INDEL densities were a general feature of pericentromeric regions present in all sorghum chromosomes (Figs. 2, 4).

**Figure 5:**
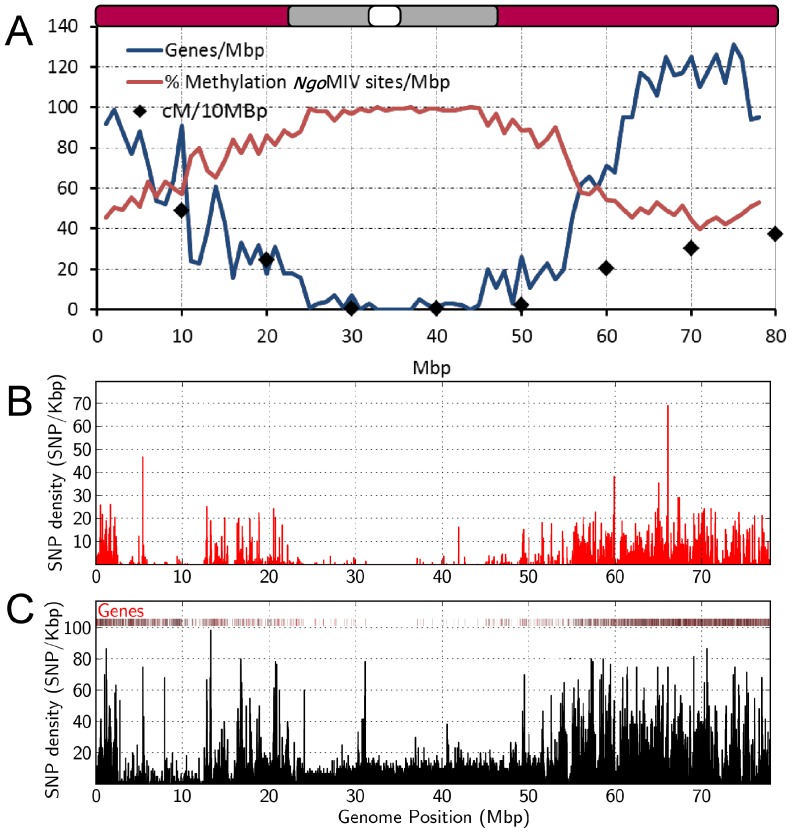
Variation in gene density, DNA methylation, recombination frequency, and SNP density across sorghum chromosome 2. (A) Gene density (blue line), percentage *Ngo*MIV site methylation (red line), and recombination (cM/Mbp, black diamonds) of SBI-02 are shown. Approximate location of the centromere (white), distribution of euchromatin (maroon), and heterochromatin (grey) are indicated at the top of the graph. Centromere location and distribution of euchromatin, and heterochromatin were obtained from Kim et al. [19]. SNP density between Tx7000 and BTx623 on sorghum chromosome 2 is shown within (B) genic DNA and (C) intergenic DNA on a SNP/100 Kbp basis. Gene density is represented as a bar graph at the top of Fig. 5C.

The relationships between SNP density, gene density, recombination and DNA methylation were examined across SBI-02 to better understand the origins of the observed variation in DNA polymorphism density distribution in different regions of this chromosome (Fig. 5). A diagram representing the molecular cytogenetic architecture of SBI-02 characterized by Kim et al. [19] is shown at the top of Figure 5. The centromere is located at ∼33–34 Mbp, flanked by heterochromatin spanning the region from ∼24–47 Mbp, and with euchromatin comprising the remaining portion of each chromosome arm. Gene density per Mbp was estimated based on gene annotations in Phytozome [37]. The highest gene density occurs in euchromatic DNA located in the terminal ∼24 Mbp and −30 Mbp portions of SBI-02, decreasing to low levels in the pericentromeric portion of the chromosome (Fig. 5A, blue line). Recombination frequency across SBI-02 was estimated using the 120 cM genetic map of this chromosome, generated based on recombination events identified in the BTx642 x Tx7000 RIL population used for genetic map construction (Fig. S1). Recombination frequency was highest in euchromatin located toward the ends of this chromosome, and lowest in the central ∼20 Mbp pericentromeric region (Fig. 5, black diamonds, cM/10 Mbp). Variation in the extent of DNA methylation across SBI-02 was analyzed by comparing the number of DG-sequences obtained from deep sequencing to the total number of DG-sequences (predicted *in silico*) that could be obtained if all of the *Ngo*MIV recognition sites used to generate DG-template were unmethylated. *Ngo*MIV is a methylation sensitive restriction enzyme; therefore DG-sequences are only generated adjacent to recognition sites that are not methylated. As expected, very few of the DG-sequences identified *in silico* in the pericentromeric region were recovered by deep sequencing DG-templates, indicating that a high percentage of the *Ngo*MIV sites were methylated. The percentage of the *in silico* predicted DG-sequences recovered by sequencing increased in euchromatin, reaching 40–50% in DNA near the ends of SBI-02, consistent with reduced levels of DNA methylation (Fig. 5A, red line).

**Figure 6:**
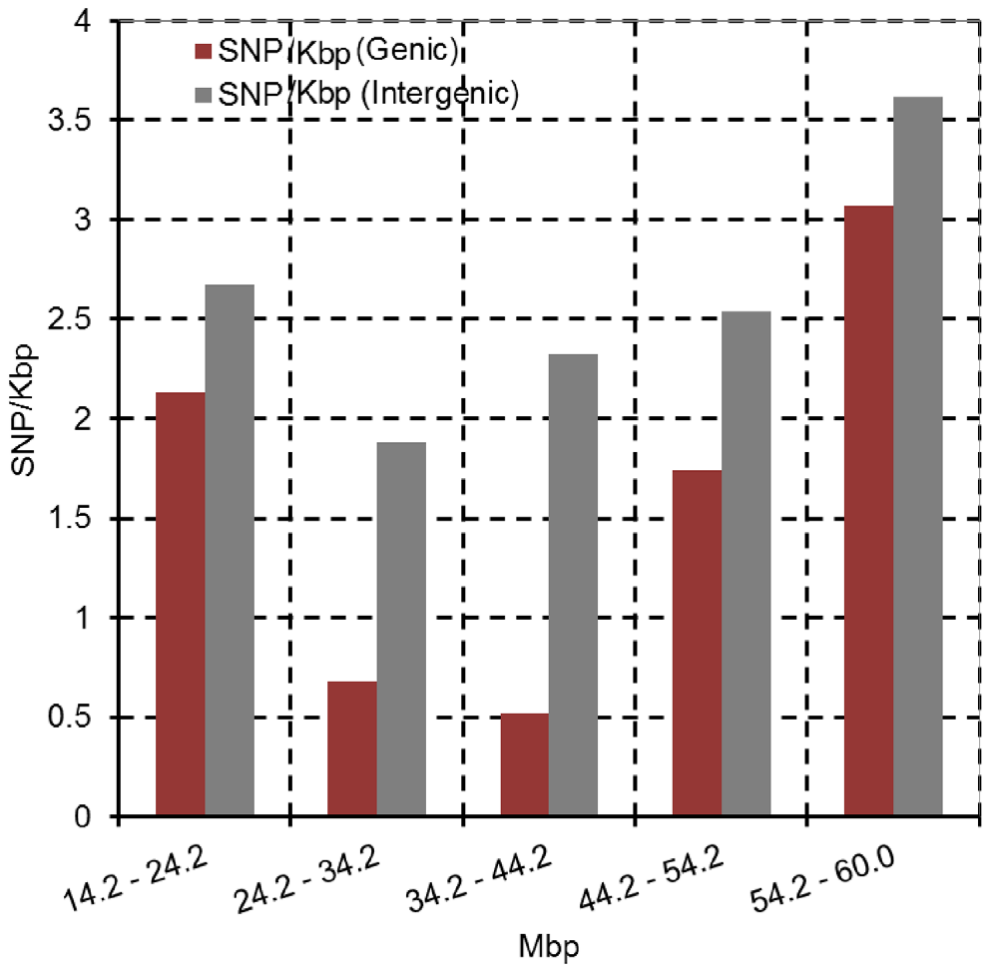
SNP density distribution on sorghum chromosome 2. Average density of SNP/Kbp within genic DNA (red) and intergenic DNA (grey) on SBI-02 in the pericentromeric region (24–44 Mbp) well as DNA flanking this region from 14–24 Mbp and 44–60 Mbp. Bins are 10 Mbp.

Variation in SNP density in each gene encoded in different regions of SBI-02 was plotted in Figure 5B (SNP/Kbp/gene). This analysis revealed elevated levels of, and extensive variation in, SNP density in genes located in euchromatic DNA, and lower SNP density in genes encoded in heterochromatic pericentromeric DNA. When total SNP density (genic and intergenic) was plotted using 600 bp windows, a similar trend was observed (Fig. 5C). Average SNP density in genic and intergenic DNA was analyzed in various regions of SBI-02 where Kafir and Durra DNA could be compared. DNA from Blackhull Kafir spans the region from ∼12.5 Mbp on the short arm of SBI-02, through the pericentromeric region, to ∼60.2 Mbp on the long arm in BTx623. In contrast, Tx7000 has DNA from Milo (Durra) across the same region. Overall SNP density in genic DNA in the pericentromeric region of SBI-02 from 24–44 Mbp was low (∼0.5 SNP/Kbp) as compared with intergenic DNA in this region (∼2 SNP/Kbp) (Fig. 6). However, DNA immediately flanking this central region showed higher average SNP density in both genic DNA (1.8–3 SNP/Kbp) and intergenic DNA (2.5–3.5 SNP/Kbp) (Fig. 6). SNP density in genic and intergenic DNA ranged from 1.8–2.0 SNP/Kbp in comparisons of Milo-Durra to Blackhull Kafir DNA across another euchromatic region of SBI-06 (46–51 Mbp) (data not shown).

### Distribution of SNP Density in Euchromatic DNA

**Figure 7:**
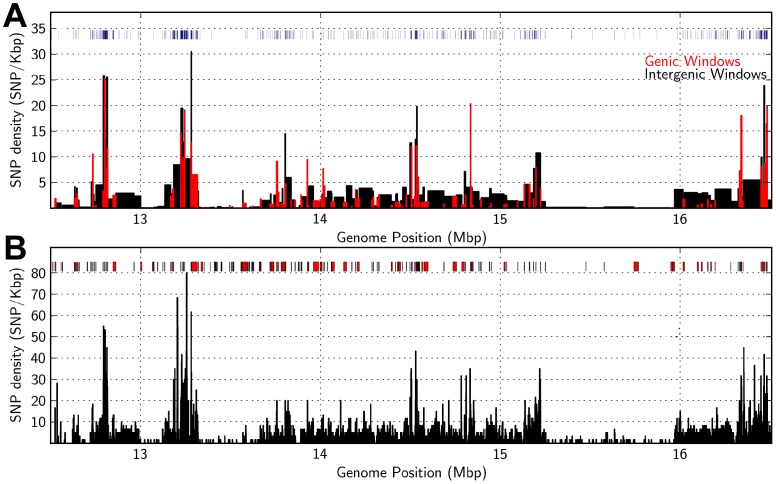
SNP density distribution across a 4 Mbp region of sorghum chromosome 2. Graphs show variation in the density of DNA polymorphisms that distinguish Tx7000 and BTx623 in SBI-02 from 12.5–16.5 Mbp. (A) Distribution of SNP density in genic DNA (red) and intergenic DNA (black) (bins of variable size, defined as the length of the genic or intergenic window). The locations and sizes of INDELs are indicated (blue, top of panel, widths of bars indicate INDEL sizes). (B) Distribution of total SNP density (600 bp bins) in SBI-02 from 12.5–16.5 Mbp. The location of genes across the 4 Mbp region is shown in a bar graph at the top of Fig. 7B.

**Figure 8:**
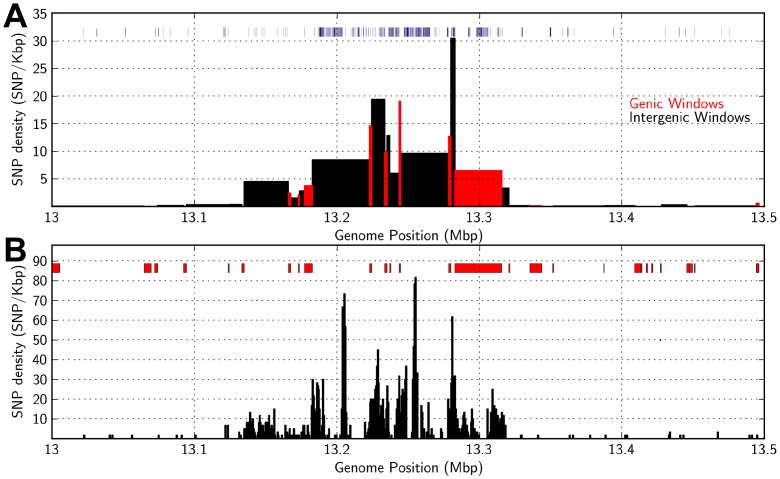
Highly variable distribution of DNA polymorphism within a 500-02 (13–13.5 Mbp). (A) Average density of SNPs that distinguish Tx7000 and BTx623 (bins of variable size, defined as the length of the genic or intergenic window) in genic DNA (red) and intergenic DNA (black). The locations and sizes of INDELs are indicated (blue, top of panel, widths of bars indicate INDEL sizes). (B) Distribution of total SNP density (600 bp bins) from 13–13, 5 Mbp across SBI-02. The location of genes in the region is shown at the top of Fig. 8B.

The SNP density distribution in a region of euchromatic DNA from 12.5 Mbp to 16.5 Mbp on SBI-02 (Kafir vs. Durra haplotypes) was analyzed at higher resolution to further characterize the basis of variation in SNP density (Figures 7 and 8). A total of ∼172 genes were annotated in this 4 Mbp region, with an average gene density of ∼1 gene per 23 Kbp. Genes were distributed across the entire region with varying densities, ranging from 1/31 Kbp (13–13.1 Mbp), 1/14 Kbp (13.3–13.7 Mbp), to 1/140 Kbp (15.3–15.9 Mbp) (Fig. 7). Plots of total SNP density assayed at 600 bp intervals, revealed several regions of high SNP density (∼40–80 SNP/Kbp at ∼12.7 Mbp and 13.2–13.3 Mbp). Other DNA intervals showed SNP densities of 10–20 SNP/Kbp (14–14.5 Mbp), and one region, which spans ∼600 Kbp from 15.3 to 15.9 Mbp, showed <1 SNP/Kbp (Fig. 7B). Average SNP density in each gene and intergenic region of DNA was analyzed and plotted (Fig. 7A). INDEL density also changed in parallel with SNP density (Fig. 7A, density bar graph). This indicates that SNP and INDEL density in genes and intergenic DNA increased and decreased in parallel in regions of varying size, but often spanning 25–300 Kbp.

The region showing the highest SNP density in this 4 Mbp region of euchromatic DNA is centered at ∼13.25 Mbp on SBI-02. This region spans ∼180 Kbp and encodes ∼10 genes (Fig. 8). In this 180 Kbp region, SNP density was elevated in both genic and intergenic DNA (Fig. 8A). When all SNPs across this 180 Kbp region were plotted, four peaks of high SNP density were identified, each located in an intergenic region (Fig. 8B). Intergenic DNA in this same interval showed elevated numbers of INDELs as well (Fig. 8A, density bar graph). Two of the 10 genes located in the 180 Kbp region were annotated as encoding hypothetical proteins in BTx623 (Sb02g009240, Sb02g009250). Deletions were present in exons of both predicted genes in Tx7000. The other 8 annotated genes in this region of elevated SNP density contained 70 amino acid differences that distinguish Tx7000 from BTx623. Most genes in the ∼180 Kbp region contained approximately equal numbers of synonymous and non-synonymous substitutions. However, Sb02g00980, a gene annotated as encoding a Major Facilitator Protein, showed a cluster of 5 non-synonymous mutations in the first exon, which may indicate that this gene is under non-neutral selection in the two sorghum genotypes. In addition to the large number of short INDELS in the region of high SNP density, there were a significant number of larger regions where no sequences from Tx7000 aligned to BTx623. Nearly all of these were in intergenic DNA and they ranged in size from 25 to ∼500 bp. The sizes and locations of these putative INDELs, plotted at the top of Figure 8A, indicate that there may be a significant difference in intergenic DNA content between genotypes in this region of high polymorphism density.

The ∼180 Kbp region of high SNP density was flanked by DNA with low SNP density (∼0.1–0.2 SNP/Kbp) spanning 90 Kbp (4 genes) and 340 Kbp (∼25 genes) (Fig. 8). Genes encoded in the flanking regions of low SNP density were nearly devoid of amino acid changes and showed low INDEL density, consistent with the overall low frequency of DNA polymorphism in these regions. Gene density in the flanking regions was similar to the region of high SNP density located between them. Differential DG-sequence recovery (vs. *in silico*) was used to assess variation in DNA methylation in the interval of SBI-02 centered at 13.2 Mbp (Fig. 8). DG-sequences recovered by sequencing were located in or near 26 of the 29 genes encoded in the region analyzed, consistent with reduced DNA methylation in genic regions. In contrast, few of the DG-sequences predicted *in silico* located in intergenic DNA were recovered, indicating that these sequences are methylated. Taken together, these analyses failed to show a correlation between variation in SNP density and gene density or DNA methylation in this region of SBI-02.

A different region of low SNP density (0.12 SNP/Kbp) from 15.3–15.9 Mbp on SBI-02 that encodes only 1 gene per 140 Kbp was also examined in greater detail. The highly conserved gene encoding glutamate decarboxylase (Sb02g010470) was located in the middle of this region of low SNP density. Analysis of DNA methylation patterns revealed by differential DG-sequence recovery showed that most of the DG-digestion sites across this region were methylated, with the exception of sites near to or within the genes Sb02g010450, Sb02g010480, and Sb02g010500 (data not shown).

## Discussion

This study reports whole-genome re-sequencing of the Tx7000 and BTx642 genotypes of *Sorghum bicolor* and the discovery of ∼2.8 million SNPs and ∼300,000 INDELS that distinguish one or both of these genotypes from the reference genotype BTx623. DNA polymorphisms were predicted to result in ∼25,000 amino acid differences and ∼250 alterations in the positions of stop codons that could affect protein function in each genotype compared with BTx623. Comparison of re-sequenced sweet sorghum genomes to BTx623 also revealed high levels of genic polymorphism that could be associated with differences in traits in sweet sorghums compared with the grain sorghum BTx623 [38]. Extensive genic polymorphism rates are consistent with high rates of QTL discovery in sorghum populations derived from bi-parental crosses [39] such as the RIL population derived by crossing BTx642 and Tx7000 [23].

Comparison of the three sequenced genomes, all of which were derived primarily from six diverse sorghum accessions during breeding, revealed greater than 100-fold variation in the density of DNA polymorphisms in different regions of sorghum chromosomes. Similar variation in SNP density has been found in larger-scale studies of *Arabidopsis thaliana* ecotypes and among diverse genotypes of maize and rice [2], [40], [41]. In sorghum, the distribution of SNP and INDEL density was highly heterogeneous, especially in euchromatic DNA. A portion of the large-scale variation in SNP density was due to the presence of DNA from different sorghum races in each sequenced genome and regions of the genomes that were identical by descent. Minimal polymorphism was found in blocks of DNA that had nearly identical haplotypes and similar origins, as expected. The breeding process that led to the development of BTx623, Tx7000, and BTx642 started in the 1920’s and required several cycles of crossing and selection by U.S. sorghum-breeding programs. Digital Genotyping of lines comprising the pedigrees of the sequenced genotypes showed that DNA in each of the genomes could, for the most part, be traced back to founder lines (accessions) of Kafir, Durra and Caudatum races introduced into the U.S. starting in the late 1800’s. Several genomic regions were identical by descent in all three genotypes, such as DNA spanning the *dw1* and *dw3* loci, which confer the short plant stature typical of grain sorghum [42]. Several other regions of the genome that had the same haplotype in all three genotypes could encode genes or alleles conferring early flowering, reduced seed shattering, pest resistance, improved grain yield, or other traits under selection by breeders.

Significant variation in the distribution of SNP and INDEL density across chromosomes was present after accounting for haplotype structure and regions of DNA that originated from the same founder lines or sorghum races. In general, higher SNP and INDEL densities were present in euchromatic chromosomal regions located closer to the ends of chromosomes compared with heterochromatic pericentromeric DNA consistent with results from genotyping by sequencing analysis of 971 sorghum accessions [36]. Pericentromeric regions are comprised primarily of repetitive DNA that is heavily methylated. These regions span ∼20 Mbp (e.g. SBI-02) of DNA on each sorghum chromosome, are gene poor, and exhibit very low rates of recombination [19], [28]. SNP frequency in genic DNA located in the pericentromeric region of SBI-02 was ∼0.5 SNP/Kbp, lower than in intergenic DNA located in this same region of the genome, and lower than average SNP density in genic DNA in regions of euchromatin (∼1.8–3 SNP/Kbp). The observation of low SNP density in genes present in pericentromeric regions that exhibit low recombination is consistent with prior observations in *Drosophila melanogaster*, where ‘background selection’, or the elimination of deleterious mutations in regions of low recombination, was shown to reduce variation in these regions [43]. The higher relative SNP density in intergenic DNA compared with genic DNA within pericentromeric regions could be due to higher rates of mutation in repetitive DNA due to transposon insertion, unequal homologous recombination, and illegitimate recombination [44]. In addition, lower accuracy of SNP calling in highly repetitive DNA present in the repeat-rich pericentromeric DNA could contribute to the observed difference in SNP densities between genic and intergenic DNA in these regions.

Extensive variation in SNP and INDEL density distribution was found in euchromatic DNA on every chromosome. Regions of very high SNP density (∼40–80 SNP/Kbp) alternated with regions of medium or low SNP density (<0.5 SNP/Kbp) of varying size. In one 4 Mbp region on SBI-02, there were two regions of high SNP/INDEL frequency, three regions of low SNP/INDEL density, and one extended region of intermediate SNP/INDEL density. A 600 Kbp region on SBI-02 (15.3–15.9 Mbp) of low SNP and INDEL density was gene poor and showed a high level of DNA methylation, similar to heterochromatic DNA in pericentromeric regions. This region of low SNP and INDEL density could correspond to a localized domain of heterochromatin within a larger region of euchromatic DNA, as observed in cytogenetic analysis of sorghum chromosomes [19]. No recombination events were observed within this region of low SNP density in the RIL population derived from BTx642 and Tx7000. Selection for glutamate decarboxylase that is encoded in this 600 Kbp region could have contributed to the low density of DNA polymorphism.

Euchromatic DNA also contained regions of relatively high gene density that showed very different SNP and INDEL densities. When regions of the genome differing in haplotype were compared, SNP densities in genic and intergenic DNA increased and decreased in parallel across the regions of high SNP density, consistent with regional differences in mutation rate and/or selection and hitchhiking which affect these regions [45]. In the regions of high SNP density, INDELs ranging from 1 bp to ∼500 bp were identified, suggesting that over time DNA loss is occurring at a substantial rate in one or both genotypes being compared. INDELs of similar size were identified in comparisons of genome sequences of *Indica* and *Japonica* rice genotypes [44]. Nearly all of the large INDELs were found in intergenic DNA, although two occurred in introns as well. Removal of repetitive DNA from plant genomes is substantial and it has been postulated that differences in gene density in euchromatin and heterochromatin may be caused principally by differences in the rates of DNA loss from the two regions [44]. The prevalence of regions of high INDEL density in localized regions of euchromatic DNA is consistent with this suggestion. Variation in DNA methylation detected using the methylation-sensitive enzyme *Ngo*MIV used for Digital Genotyping showed that low DNA methylation was highly correlated with DNA located in or near genes. Since gene density within and flanking the regions of high SNP density were similar, DNA methylation was not highly correlated with SNP density, except in regions of low gene density and low SNP density associated with heterochromatin.

The variable distribution of SNP density in euchromatic regions observed in sorghum has been reported in several other plant genomes [2], [3], [40]. In sorghum, some of the variation in SNP density distribution among the grain sorghum genotypes analyzed could be due to retention of small blocks of DNA that were present in landraces selected during domestication. It is thought that early versions of domesticated sorghum were developed >4,000 years ago, then moved along trade routes and outcrossed with wild sorghums adapted to different parts of Africa, giving rise to the different races of domesticated sorghum [14]. Alternatively, or in addition, the regions of high SNP density could correspond to regions containing genes under divergent selection, or recombination hot spots that result in elevated localized mutation rates [46]. Mutation rates vary by ∼100-fold in different regions of nuclear genomes [47]–[49]. Variation in mutation rates within genomes has been correlated with varying rates of recombination [46], CpG sequences, nucleosome occupancy, transcription activity, di- or trinucleotide repeats, and homopolymer sequences [48], [50]. Recombination hot spots appear to be a significant driver of mutation rates due to the potential for errors associated with repair of double-strand breaks that initiate recombination [46]. If regions of high SNP density in sorghum correspond to recombination hot spots, this would help explain why linkage disequilibrium varies in an irregular manner across sorghum chromosomes [36]. More importantly, mutation frequency affects the rate at which gene expression/activity can change. Localization of genes for disease resistance or adaptation to the environment in or near regions of the genome with elevated mutation rates could provide a selective advantage, and these genes are often associated with recombination hot spots [2], showing the greatest variation in genome re-sequencing surveys [3], [38]. For breeders developing hybrid sorghum crops, knowledge of the heterogeneous nature of diversity in gene-rich euchromatic regions, and the presence of low genetic diversity and low recombination rates in heterochromatic pericentromeric regions, could contribute to the design of breeding approaches that accelerate crop improvement.

## Materials and Methods

### Plant Materials and Sequence Generation

Tx7000 and BTx642 seedlings were grown in a growth chamber (14 hr light/10 hr dark) under hydroponic conditions in 1x Hoagland’s solution. To minimize plastid DNA contamination of nuclear DNA, root tissue was used for genomic DNA isolation. Root tissue was collected eight days after seedling germination. The tissue was washed in distilled water and surface sterilized for five minutes in a solution containing 2.1% (v/v) sodium hypochlorite, 0.25% (v/v) Tween-20 and 1% (w/v) NaCl [51]. After sterilization the tissue was washed with 10 changes of distilled water prior to DNA isolation. Genomic DNA was isolated using the FastPrep Extraction kit and FastPrep24 Instrument (MP Biomedicals LLC, Solon, OH, USA), according to the manufacturer’s specifications. DNA template preparation and sequencing on an Illumina HiSeq2000 were carried out by the National Center for Genome Research. All sequences have been deposited with NCBI SRA under the following identifiers: BioProject PRJNA189453, Accession SRP019171. The sequences are grouped into two BioSamples within the BioProject: SAMN01942195 contains sequence information for BTx642, and SAMN01942194 contains sequence information for Tx7000. DNA used for Digital Genotyping was isolated from leaf tissue of five or more plants per genotype of 10–14 day old seedlings using Fastprep technology. Digital Genotyping template libraries were prepared according to the method of Morishige et al. [21].

### Read Alignment and Variant Detection

The BTx623 sorghum genome was used for mapping and variant detection, and was downloaded from Phytozome (version Sbicolor-79) [20], [37]. BTx642 and Tx7000 single- and paired-end sequences were aligned to the BTx623 reference genome using the *Map Reads to Reference* function of CLC Genomics Workbench 4.9 (CLC Bio, Cambridge, MA, USA). Mismatch costs were set to 2, and insertion and deletion costs set to 3. Reads were required to align for at least 50% of their length, with similarity higher than 70%. Reads that could not be uniquely aligned were discarded. SNP and INDEL detection were performed using the *SNP Detection* and *DIP Detection* functions of CLC Genomics Workbench 4.9, respectively, on the merged paired-end and single-end read alignments. Minimum coverage for a variant call was established at 5, and maximum was set at 150. The very few heterozygous loci found in these genotypes were not included in the analysis. Allele frequency over 75% for a given locus was considered sufficient to call that locus homozygous. Large INDELs were identified as sequences with zero read depth in either BTx642 or Tx7000 spanning sequences in BTx623. Annotations for the sorghum genome were downloaded from Phytozome (version Sbi1.4), and were used to determine the number of coding variants present in the two genotypes sequenced.

### Sequence Error Rate Evaluation

A BLAST database was created using the consensus sequences derived from the alignment of sequence reads from BTx642 and Tx7000 to BTx623. DG-marker sequences (72 bp read length,1,462 from each genotype) that were validated by genetic mapping were compared with this database using the blastn program integrated into CLC Genomics Workbench, with an e-value maximum of 1e-10, mismatch cost of 2, gap initiation cost of 5 and gap extension cost of 1. Marker sequences from Tx7000 (1,356 of 1,462) and BTx642 (1,293 of 1,462) aligned perfectly to their respective consensus sequences. Sequences that did not align perfectly with validated DG-sequences contained insertions or deletions that were not detected during analyses of read alignments. All SNPs within DG-marker sequences were correctly identified.

### Sorghum Lineage Tracing, Genetic Markers and Genetic Map Construction

Genetic markers used to determine lineage were identified and analyzed by Digital Genotyping as described [21]. The DG genetic linkage map was constructed using genotypes assigned by analysis of sequence-based markers from 90 RILs derived from the BTx642 × Tx7000 RI population. Initial marker order was predicted based on alignment of DG marker sequences to the BTx623 reference genome sequence [20]. Recombination frequencies of DG markers were determined using Mapmaker/EXP ver. 3.0b [52]. The command ‘map’ was used to calculate genetic distance between markers, using the Kosambi mapping function. When two or more DG markers mapped to identical locations, all but one of the markers were removed prior to the next step in mapping. The order of the remaining DG markers was confirmed using ‘order’ and ‘ripple’ functions. DG markers with LOD scores >3.0 were retained in the final DG genetic map. DG markers that distinguish genotypes comprising pedigrees of BTx623, BTx642 and Tx7000 were ordered based on alignment of DG sequences to the BTx623 reference sequence.

### Dendrogram Construction

The evolutionary history of sorghum genotypes was inferred using the UPGMA method [53]. The optimal tree with the sum of branch length = 2.66637274 is shown. The percentage of replicate trees in which the associated taxa clustered together in the bootstrap test (1000 replicates) is shown next to the branches [54]. The tree is drawn to scale, with branch lengths in the same units as those of the evolutionary distances used to infer the phylogenetic tree. The evolutionary distances were computed using the Tamura-Nei method [55] and are in the units of the number of base substitutions per site. The analysis involved 14 nucleotide sequences. Codon positions included were 1st+2nd+3rd+Noncoding. All ambiguous positions were removed for each sequence pair. There were a total of 19910 positions in the final dataset. Evolutionary analyses were conducted in MEGA5 [56].

## Supporting Information

Figure S1 **Genetic map based on the BTx642 x Tx7000 RIL population and marker information derived from Digital Genotyping.** The genetic map is based on information derived from 1462 DG markers scored in 90 RILs resulting in a genetic map of 1130 cM spanning the ten sorghum chromosomes (1–10). Genetic map distances are shown to the left of each linkage group/chromosome.
(TIF)

Figure S2 **Comparison of SNP density distributions from independent sequence assemblies of two genotypes.** DNA from Tx7000 and BTx642 were independently sequenced and reassembled across SBI-02 from 75–76 Mbp using the BTx623 reference sequence. DNA spanning this region of SBI-02 in both genotypes has the same haplotype as BTx3197. SNP density analyzed in 600 bp windows of Tx7000/BTx623 (upper) and BTx642/Tx7000 (lower) generated similar SNP distribution profiles.
(TIF)
