# Supplementary figures and images for: Extensive Variation in the Density and Distribution of DNA Polymorphism in Sorghum Genomes
Source: PLoS One. 2013 Nov 12;8(11):e79192. doi: 10.1371/journal.pone.0079192 (PMC3827139; doi:10.1371/journal.pone.0079192)

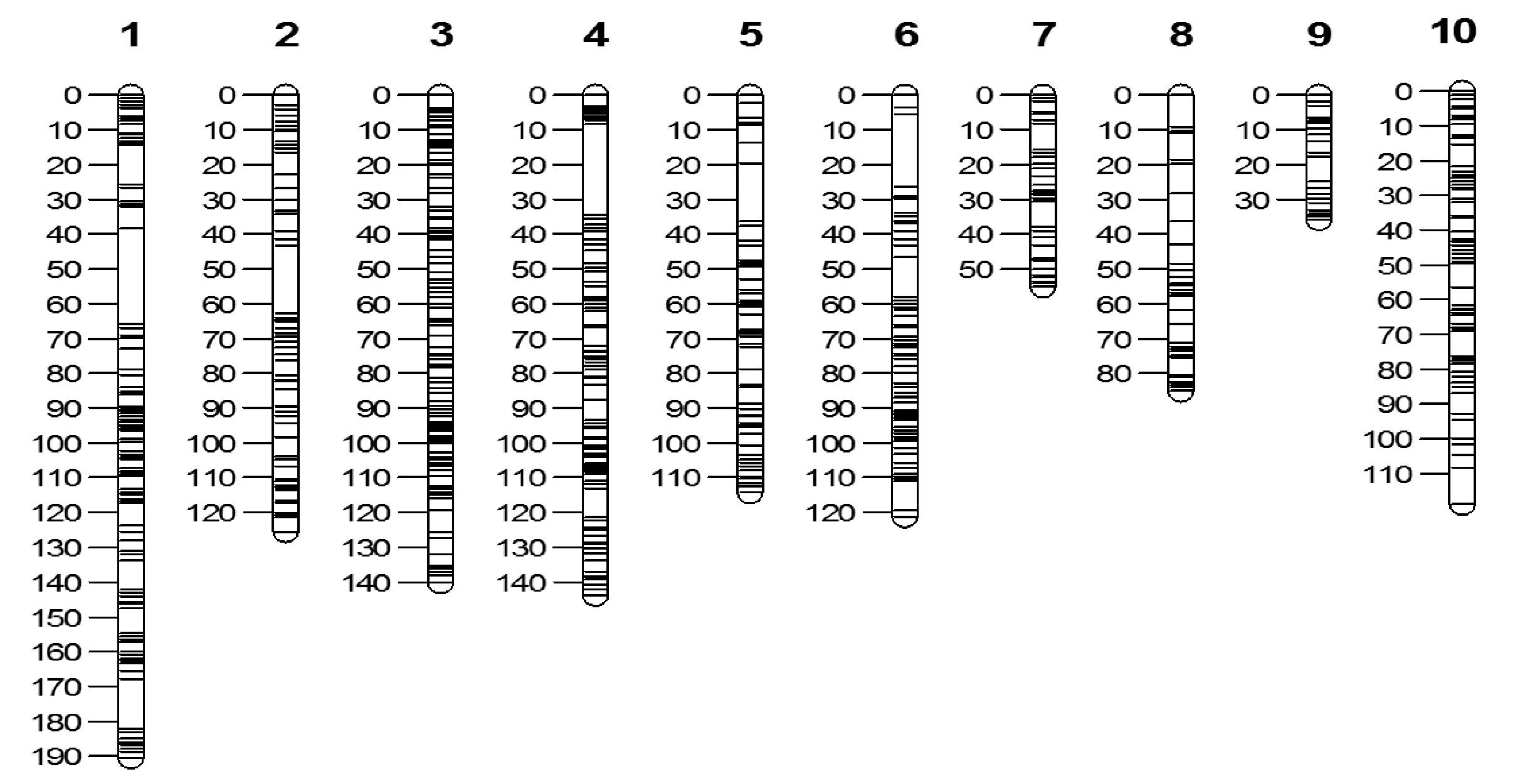

Supplement: Figure S1 — Genetic map based on the BTx642 x Tx7000 RIL population and marker information derived from Digital Genotyping. The genetic map is based on information derived from 1462 DG markers scored in 90 RILs resulting in a genetic map of 1130 cM spanning the ten sorghum chromosomes (1–10). Genetic map distances are shown to the left of each linkage group/chromosome. (TIF) [file pone.0079192.s001.tif]

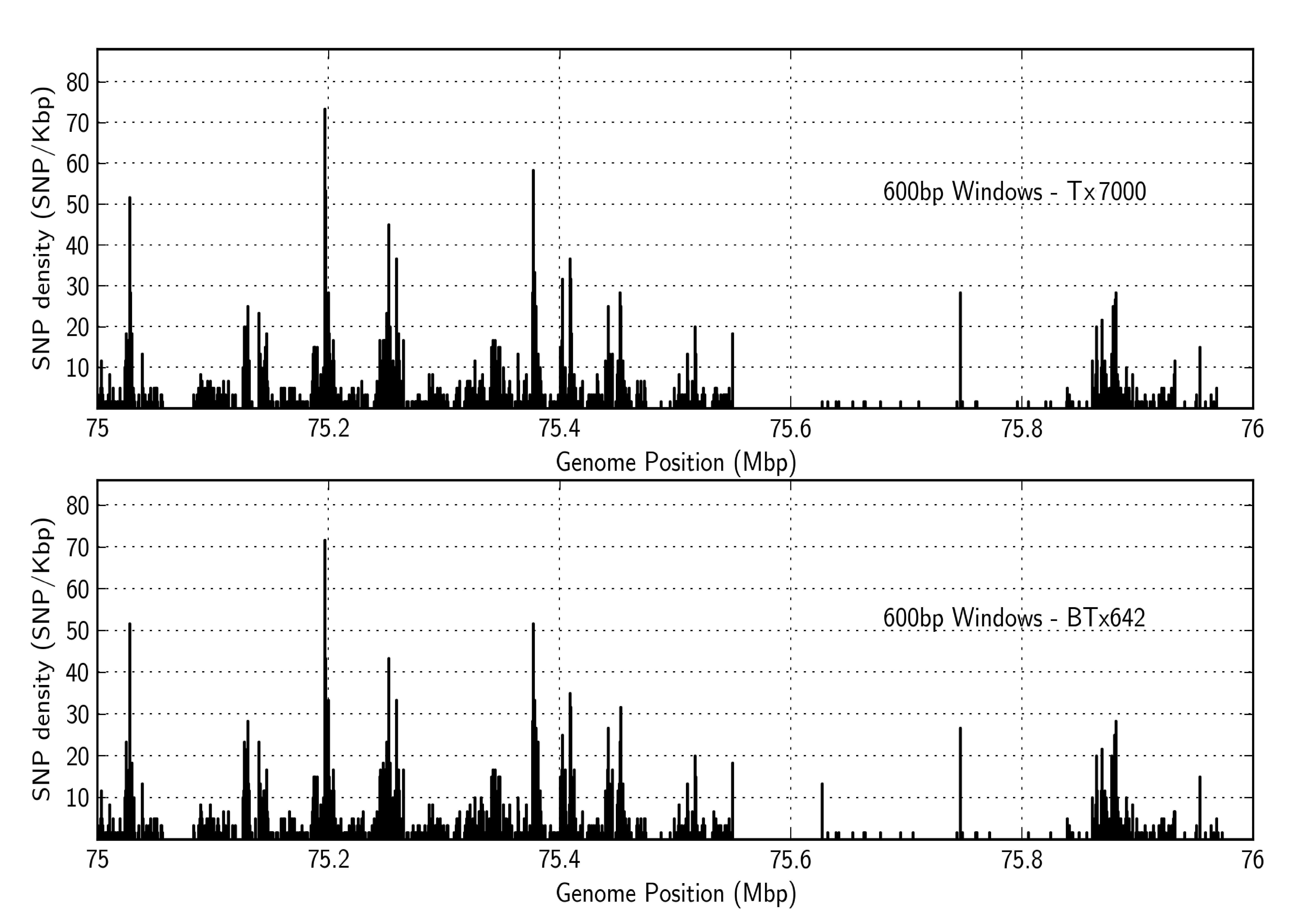

Supplement: Figure S2 — Comparison of SNP density distributions from independent sequence assemblies of two genotypes. DNA from Tx7000 and BTx642 were independently sequenced and reassembled across SBI-02 from 75–76 Mbp using the BTx623 reference sequence. DNA spanning this region of SBI-02 in both genotypes has the same haplotype as BTx3197. SNP density analyzed in 600 bp windows of Tx7000/BTx623 (upper) and BTx642/Tx7000 (lower) generated similar SNP distribution profiles. (TIF) [file pone.0079192.s002.tif]
